# Address, Introductory to the Sixth Course of Lectures in the Atlanta Medical College

**Published:** 1860-06

**Authors:** J. G. Westmoreland

**Affiliations:** Professor of Materia Medica, Therapeutics, and Medical Jurisprudence


VOL. V.] JUNE, 1860. [NO. 10

### ORIGINAL COMMUNICATIONS.

### ARTICLE I.

*Address, Introductory to the Sixth Course of Lectures in the Atlanta Medical College*, by J. G. WESTMORELAND, M. D., Professor of Materia Medica, Therapeutics, and Medical Jurisprudence.

GENTLEMEN OF THE MEDICAL CLASS:

I have been honored by the Faculty with the pleasing duty of welcoming you to the halls of the Atlanta Medical College; and not only to the halls of science, but to the society of the citizens of Atlanta.

It is not here as at some other points where Medical Colleges are located: the student in Atlanta is recognized as a human being—a man—aye, a respectable gentleman—and as such is admitted into that circle which the profession he has chosen requires him to move in through after-life.

It is true that here, as at other places, where conduct measures respectability, those whose acts render them unworthy the confidence and respect of the community, are placed in their proper sphere, with whatever calling they may be connected. But, to the praise of the classes which have preceded you, be it said, as a body, no class, profession or calling affords a smaller proportion addicted to habits of immorality than they; and to this fact, perhaps, more than any other, is attributable the unexampled respectability of the student here. Not only so, but the Institution is indebted to the moral deportment of the high-toned gentlemen composing the former classes, in a good degree, for the character it has, and for the number of intelligent faces before me to-day.

No Faculty labors more assiduously, it is true, to build up and sustain the reputation of their school, by directing their whole energies to the thorough instruction of their pupils, yet, without the proper kind of material, all their efforts would be futile. The moral character, intelligence and noble bearing of the alumni of an institution add greatly to its character. "By their fruits ye shall know them," is considered by many no less true of institutions of learning than when applied to individuals in a moral or religious sense.

We welcome to-day the sixth class to our Halls, and in no vain-glorious or boastful spirit would we say, that from the first, which met in 1855, to the present there has been a steady increase of the number in attendance every year. We feel more and more determined, with every such demonstration of approval of our labors, to spare no pains to make the Course such an one as will be most profitable and interesting.

Gentlemen, suffer me to say again, welcome, thrice welcome to our city, to our Halls—and, from the greeting which we meet from the many fair faces on this occasion, I may add, and to the society of the gentler sex also.

We offer you a temporary residence in a city of unequalled healthfulness. On the dividing ridge between the waters of the Gulf and the Atlantic, away from localities of pestiferous malaria, Atlanta is a fortunate selection for a Summer Medical College. We promise you more than ordinary health, comfort, and the right hand of all good citizens; many of whom, to their praise be it said, have thrown open their doors and made boarding-houses of their heretofore retired private residences—moved by a desire for the continued prosperity of the institution, and by the interest felt in those in attendance upon its exercises. It is true, however, to those accustomed to the comfort of a quiet village, or the pleasures of retirement on a farm, the bustle of our Railroad City—the neighing and ponderous tread of the iron horse, as he sweeps through the streets with his thousands of commerce, may for a while prove a source of annoyance and attract the mind from the laborious duties that lie before you, but these difficulties pass away with the familiarity of such sounds, which is soon acquired.

To-day we open you the doors of a Summer School of Medicine, the only one in the South, at the time of its organization—the pioneer in the system of summer teaching south of Philadelphia. It was then called a useless experiment by those north of us, who, in medical teaching as in commerce, grow rich upon Southern contributions. And while they court patronage to sustain their mercantile establishments and institutions of learning, the most fiendish denunciations are pronounced politically.

While the theory of Medicine is the same North and South, and while facilities for learning have not diminished, recent demonstrations of political demagogues and abolition fanatics North have determined a portion of their patrons South to withdraw support in any way from a people continually waging political warfare against them. They determine no longer to give strength, power and influence to a section whose highest ambition is our political and commercial demolition.

Now, while we would not detract in the slightest degree from the deserved reputation of Northern Medical Colleges, it is evident, from the aspect of political matters, we should be admonished to look well to home institutions. It is necessary that we be prepared in summer as well as winter to supply the demand of the medical learner with all the facilities necessary to acquire a medical education when it is his preference to accept it. This we are attempting to do, and it is for you, and others who may visit the institution, to determine whether or not that attempt has been successful.

In addition to the several Winter Schools in the South and West, there are now two regularly organized Summer Medical Institutions, holding but one regular session during the year. One of these in Atlanta, Georgia, and the other in San Francisco, California. North, in addition to the Winter and quasi Summer Schools, there is in Brooklyn, N. Y., a bona-fide Summer Medical College, recently established. Of which an eminent medical man of New York City, in a recent letter to me, says: "Your success in the Summer Course has resulted in the Brooklyn Summer School, has disarmed opposition."

From this we would infer that opposition to our enterprise had once existed, and that it arose either from jealousy or a want of confidence in its practicability; and also that it has been allayed by other proofs of the possibility of success with Summer Schools, or the addition of other influences to secure popular favor.

The Summer Schools established, seem at present sufficient to the demand for summer teaching. If others become necessary there is no doubt they will be organized; and should new institutions be founded, it will not be our pleasure to throw difficulties in their way.

In no spirit of rivalry, nor with a disposition to pull down others for our own promotion, was this institution established. This feeling does not yet exist in the breasts of those who control its movements; and when from jealousy it becomes the policy of the Atlanta Medical College to detract from the character of others, in order to increase her own numbers, she will become unworthy the position she has attained, and unworthy the confidence and respect of Medical students. Upon merit let her stand or fall.

The enterprise of Summer Medical Schools in the South, five years ago, was an untried experiment, and was undertaken in Atlanta in the face of the generally believed and oft-repeated opinion that Anatomy could not be taught practically in warm weather. To-day, no one that has any regard for his reputation as a man of information in medical matters would dare question that fact. In five years more

—mark the prediction—the opinion will be generally entertained that this important branch of medical science can be *more* profitably prosecuted during summer than winter. By this, we mean no disparagement of the Winter Course of study. No; the medical pupil can study all the departments in all seasons; and it is important to him that facilities be afforded winter and summer. The time which is spent in the usual office study, between the regular Courses, confined to one season, consisting of about eight months, is, to say the least of it, unprofitable in preparing the student for the duties of the profession.

Success, we say, then, to all Medical Schools that merit success, by affording the proper means for the acquisition of scientific knowledge, whether in winter or summer. And if any there be that fail to come up to the curriculum of studies usually taught; that confer the Degree upon unworthy applicants, who are a disgrace to the profession, and a bain to the community; or that, by unjust inuendoes and otherwise, seek to defame the character of other institutions for their own aggrandizement, it needs not our condemnation to direct attention to their conduct, nor our accusation to arraign them before the bar of public opinion.

The history of Summer Schools at the time of our organization, was not at all encouraging to the undertakers of the enterprise. So unsuccessful had summer teaching uniformly proven to be in the North and West that failure was confidently predicted by our enemies, while serious fears were entertained by many who wished the project success. And not until the test of time had blasted the hopes of the one and calmed the fears of the other, was the significant fact taken into the account, that most of the unsuccessful efforts of the kind were made in connection with Winter Schools, and conducted by the same teachers.

When two full Courses are attempted during the year by the same Faculty, the proper facilities are not usually afforded to warrant success in both. And, again, the practice pursued by some of these institutions of granting examinations for the Degree at both sessions was not only looked upon as of questionable propriety, but openly condemned by a large proportion of the profession.

One of the most universally acknowledged evils in medical education is the conferring the Degree upon those not prepared to assume the duties of the profession. The various remedies of lengthening the term of lectures, of increasing the time for preliminary office study, of requiring a certain time between the Courses of Lectures, &c., &c., have been suggested, but after all the plans proposed shall have been carried out, the only dependence at all reliable is upon the requisitions for graduation. And if teachers would confine themselves to their own legitimate province of teaching properly, and confer the Degree on none but those learned in the science, and let students study when and where they please, the great *desideratum* would be attained. No particular length of time spent in study, previous to examination, by any means insures thorough preparation, but when adopted serves as a scape-goat for the sins of teacher and pupil. Two years of nominal study may not be worth two months of laborious investigation, and the requiring of eight months interval between the Courses taken, would only allow the student more perfectly to forget what he had already learned. Again, by making time of study the test of qualification, the one important incentive to study is lost, and the restless candidate for the honors of the Degree occupies himself in making time pass swiftly and pleasantly away.

Next in importance to the determination on the part of the student himself to learn, is the manner of teaching, in order to the rapid progress of the pupil. When he has to be engaged in the consideration of several subjects during the day, it is necessary that they be passed in review frequently, in order to their permanent impression upon the mind. To this end, the system of *examination* is an established usage with us. At each lecture, the class, or so many as desire it, are questioned on the subject of the previous one, thereby fixing more permanently the points considered. Then we conclude, that to the improvements in the manner of teaching, and other facilities for imparting instruction, must we look for the rapid progress of the learner, and to the standard of green-room requirements for protection to the profession and to the community against poorly qualified physicians. This is the doctrine we profess. This is the doctrine we practice.

Now, gentlemen, let me direct your minds to some of the difficulties and self-sacrifices, as well as the pleasures which are to be met with in acquiring a knowledge of the profession of your choice.

It was said by a philosopher, "The proper study of mankind is man." This is particularly true when applied to the physician. The study of man is the Alpha and Omega—the theme paramount to all others that will engage your attention. Man, Anatomically, Physiologically, Pathologically and Psychologically, comprise studies so intricate, so profound so extensive, that minds the most active and energetic have been worn out without fathoming all the depths of these vast subjects. Every branch of medical science is either directly explanatory of the human organism, includes the study of some of the phenomena of man's existence, or teaches the means of perpetuating it, when in jeopardy from disease or accident. Not only so, but the study of the nature of mankind, so as to enable you to please, influence and control, is perhaps more important to the physician than to any other profession. These, with the study of the laws that govern inorganic and vegetable matter, and their therapeutical and physiological adaptation to our race, make up an array of studies calculated to frighten the irresolute student of medicine from his purpose. Think not that your pathway is strewn with roses, and that your pupilage will be made up of ease and pleasure. He who so concludes, deceives himself, or has made up his mind to have "the form without the matter,"—to get the title without a knowledge of the science. In either case, I would say to such, select some other calling.

Nor is the picture bounded here. The vast responsibility devolving upon those taking upon themselves the duties of the profession, makes it incumbent upon the student to lay well the foundation of his studies by a thorough knowledge of those fundamental scientific truths, without which the superstructure of the more practical branches will be as houses with foundations of sand, that totter, tremble and fall under the stormy test of practical application.

Your rudimental studies must be thorough, and when the mind has grasped all the well-established principles, it is necessary that they again and again be presented to the mind in order to durable impression. The theories of ages have also to be sifted for solid material, and weighed and compared by the acknowledged facts already learned. Think not that your preliminary pupilage and Collegiate Course are to bound the time of your laborious investigations. The end of your professional career is the limit of your labors. Enter the struggle with these facts before you. Prepare yourselves to undergo the toil of unending research, and, my word for it, you will not be disappointed.

But another phase of these labors and responsibilities may be presented. Man, differing from the inferior animals, is endowed with a mind, subject to the highest degree of pleasure and happiness, and to the deepest remorse. In the exercise of the various faculties alone are these emotions produced. To the mind of scientific cast, the toil of exploring mines of hidden scientific truths is a pleasure, and in the dispensing those treasures exists one of the sources of true happiness on earth. Sometimes, it is true, when the principle sought lies deeply hidden, the task of removing the rubbish is irksome and annoying for awhile, but gives place to joys all the brighter for the darkness which preceded them.

It has been truly said that "Knowledge is power," and to this trite axiom may with propriety be added, and in the acquisition of knowledge is found true pleasure.

To him who commences the study of the medical profession with no other feeling actuating him, and with no other hope pushing him forward than the pecuniary reward it bestows, the pleasures that sweeten toil do not come. Those gems that glitter to the scientic eye, and give fresh impulse to energy and industry in the pursuit of knowledge, do not attract his attention. So with the careless observers of science, from whatever motive prompted to attempt its study, or seek the honors of the profession. Such have their only pleasure in indolence, and recreation, and can never become proficient.

It is not alone necessary that the *student* of medicine, during his pupilage, feel interested in the science beyond the honorarum in dollars and cents, but in a higher degree should the purposes of the *practitioner* be elevated above mere pecuniary considerations. The learned professions are not mere trades, to be sought by those out of employment, in order to secure a competency, but are intended to elevate the man intellectually and morally, by expanding the mind into scientific, legal and political truths from which mankind is to be benefited. The medical profession is an elevated, noble calling, intended to bless mankind with untold benefits; and while to its members ample pecuniary supplies, and sometimes abundant wealth is secured, as a part of their reward, the consciousness of having fathomed the deep mysteries of science, and in their application conferred a blessing upon the world, is superior remuneration—pleasure which gold cannot buy.

But young America, always on the alert in this fast age, asks if the route cannot be shortened, the amount of study curtailed. Visions of a panacea for the cure of every ill to which mankind is subject haunt the impatient student of medicine, while with eagerness he seeks for evidences of the unity of disease. The ill-gotten wealth of some nostrum patentee glitters in his view, and lures him from the walks of science toward peculation and humbuggery. The one-ideal systems, one after another, pass in review before him, and that which promises the shortest course of study, or the most speedy return of fees, is selected, whether it be the theory of Hahneman, Preeisnitz, or Thompson, with their respective materia medicas, sugar pills, water and pepper. 'Tis wrong. Though lost to the love of science, and willing to get gain by deception and fraud, there should be yet sufficient moral sensibility remaining to choose the system least liable to do harm—that system whose so-called remedies, in the language of the officiously benevolent dame, at the bed side of a suffering neighbor, "If they do you no good, will do you no harm;" or, which, according to the transposition of the facetious wag, and more literally true, "If they do you no harm, will do you no good."

The system first suggested by Stahl and called by him the "expectant," or "waiting system," afterwards revived by Hahneman in the form of "*Infinitessimal*" doses—now familiarly known as the "pellet" or "sugar-pill" system is, of all one-idea systems, least likely to produce direct injury.

Should you then, gentlemen, meet with any seeking a shorter route to the temple of Æsculapius (for I have no idea that any of *you* have been thus tempted from the paths of science,) please say to such, choose Homœopathy, the waiting, expecting, hoping system. For he who gives virtually nothing at all, is less culpable than the rash dealer in active remedies, of the nature and application of which he is totally uninformed.

Six-tenths of all the cases asking medical aid in absence of a malignant epidemic, would recover, finally, without any treatment at all. And although many lives are lost under the "expectant" or waiting plan, that might be saved by timely medication, yet, not only these but some of those likely to survive unaided, may be sacrificed by violent drugs in the hands of empyrics.

The following lines of the poet are more particularly true when applied to the study of the healing art, than to any other scientific or literary pursuit:

> "A little learning is a dangerous thing,
> Drink deep or taste not the Pierian spring."

Whether viewed from a moral, legal or political standpoint, the inevitable conclusion must be, that the uninformed practitioner of medicine is an evil in the whole circumference of his operations.

To the deficiency in medical learning must we look for the chief cause of the numerous pathies so rife in the land; and to the importance given the various sects, humbugs and nostrums, by those occupying high positions in the religious, legal and political walks of life, in some degree, for the limited learning in medicine so frequently found.

The cause which you are about to enter, acknowledges the name of no sect, and is governed by the motto of no exclusive principle in Therapeutics. Allopathy is no more expressive of the science of medicine than is the title of any exclusive sect; nor is the motto *contraria contrarii curantur* any more appropriate to the extensive untrammeled calling we profess. Let all the pathies go down together.

He who degrades the profession to that of a sect, by recognising the term Allopathy, and wrestles for the mastery with the one-idea systems of the day, brings upon himself the just reproach of all scientific men of extensive views.

No term is more appropriate to the Healing Art than that of Scientific Medicine; and all the sects, making pretensions to science, which have been springing up from time immemorial, are but scintillations from general medicine, magnified into universal science, in the brains of those impatient for fame, or too shallow to comprehend all the practical truths of medical science.

As a talisman, to prompt us, and all others who may enter the profession, to sustain its honor, dignity and usefulness, by discarding the names of sects, and the doctrine of exclusive principles, let all invoke, continually the names of Pereira, Hunter, Bell, Chapman, Physic, and other venerable patriarchs in medicine and surgery, whose lives were spent in sustaining the great cause, and adding new truths for our use.

Let me again insist upon a thorough knowledge of the fundamental principles in medicine, which makes the physician, and without which no one should occupy so important a station. Not less responsible than he who ministers words of peace to the soul, is the physician, who to the tortured physical frame, threatened with dissolution, gives ease, comfort, hope, and "stays the ruthless hand of the dread monster."

While the occupant of the sacred desk has to deal with the soul, the much more important part, he is guided by the chart of infallible origin, in his ministerial functions; and in the exercise of his legitimate duties in directing the conduct of mankind, has only to point them to the unerring word of truth, which he is forbidden to diminish or add to, in the slightest degree. Not so with the physician. From various, and sometimes, contradictory theories, he is often forced to make up an opinion, upon the correctness of which depends the life of a human being. Not of one who has forfeited his existence by a breach of the law of the land, but of the helpless infant, the promising youth, the worthy citizen.

So long as this responsibility is disregarded by those who are called doctors, and so long as the people generally are uninformed in the well established principles in medicine, will the brazen pretender flourish for a while in a place, whether he presents himself as the proselyte of a one-ideal system, as the inventer of a cure-all nostrum, sustained by certificates of statesmen, clergymen, &c., or in the livery of legitimate medicine.

It is the nature of the human family to be attracted by novelties. It is but reasonable to expect that intelligent minds will be impressed by glowing descriptions and flattering reports and certificates of success, even though they come from those no better informed in medicine than themselves.

Now, while the pecuniary profits of the profession, in the aggregate, are not curtailed, but often enhanced by the temporary success of interlopers; and while notwithstanding this, every effort on the part of physicians to correct the evil, is looked upon as a selfish move, I hold it to be the duty of every member of the profession, for the sake of their neighbors, for the sake of humanity, and for the moral responsibility upon him to pursue such a course as will most effectually thwart the designs of empirics.

In conclusion, let me say, that he who commences the study of medicine, with the knowledge of what lies before him, and is actuated by proper motives in the pursuit of science, will never fall a victim to the wiles of empiricism. His mind, during his pupilage will become so thoroughly imbued with the principles that govern the true physician, that his professional course will be run with profit and satisfaction to himself, honor to his calling, and benefit to his race—leaving him, at the close of his earthly career, with the consciousness of a life unsullied by ill-gotten gain—a life of rectitude toward God and man.

